# Ultrafast Fiber Lasers: An Expanding Versatile Toolbox

**DOI:** 10.1016/j.isci.2020.101101

**Published:** 2020-04-25

**Authors:** Guoqing Chang, Zhiyi Wei

**Affiliations:** 1 Beijing National Laboratory for Condensed Matter Physics, Institute of Physics, Chinese Academy of Sciences, Beijing 100190, China; 2 School of Physical Sciences, University of Chinese Academy of Sciences, Beijing 100190, China; 3 Songshan Lake Materials Laboratory, Dongguan, Guangdong 523808, China

**Keywords:** Laser, Nonlinear Optics, Fiber Optics

## Abstract

Ultrafast fiber lasers have gained rapid advances in last decades for their intrinsic merits such as potential of all-fiber format, excellent beam quality, superior power scalability, and high single-pass gain, which opened widespread applications in high-field science, laser machining, precision metrology, optical communication, microscopy and spectroscopy, and modern ophthalmology, to name a few. Performance of an ultrafast fiber laser is well defined by the laser parameters including repetition rate, spectral bandwidth, pulse duration, pulse energy, wavelength tuning range, and average power. During past years, these parameters have been pushed to an unprecedented level. In this paper, we review these enabling technologies and explicitly show that the nonlinear interaction between ultrafast pulses and optical fibers plays the essential role. As a result of rapid development in both active and passive fibers, the toolbox of ultrafast fiber lasers will continue to expand and provide solutions to scientific and industrial problems.

## Introduction

**Figure 1:**
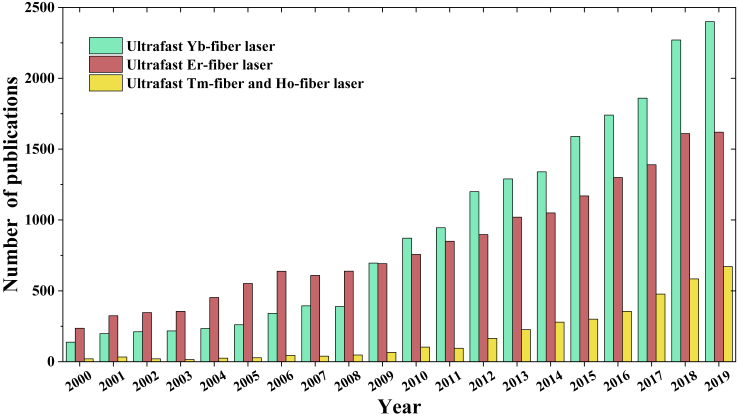
Number of Publications Versus Year from Google Scholar Key search words: ultrafast ytterbium fiber laser, ultrafast erbium fiber laser, ultrafast thulium fiber laser, and ultrafast holmium fiber laser.

This year—2020—marks the 60^th^ anniversary of the invention of laser. When Theodore Maiman invented laser in 1960, it was widely considered a “solution looking for a problem (Townes, 2002).” The past six decades have seen emergence of many types of lasers, which are grouped into different categories in terms of gain materials (e.g., gas, liquid, semiconductor, solid-state, fiber), pumping schemes (e.g., electrical pumping or optical pumping), cavity configuration (e.g., linear cavity or ring cavity), operation state (e.g., CW or pulsed), etc. As one subcategory, pulsed fiber lasers dated back to 1983 when partial mode-locking was first observed in a Nd-doped fiber laser (Dzhibladze et al., 1983). Several years later, improved mode-locking in Nd-doped fiber lasers produced picosecond or even femtosecond pulses (Fermann et al., 1990b, Wigley et al., 1990). However, research in ultrafast Nd-doped fiber lasers gradually diminished owing to the development of other active fibers with advantageous properties. These superior ultrafast fiber lasers work at three wavelength ranges; that is, ultrafast Yb-fiber lasers at ~1.03 μm, ultrafast Er-fiber lasers at ~1.55 μm, and ultrafast Tm-fiber or Ho-fiber lasers at ~2 μm. Figure 1 illustrates the number of publications as a function of year for these ultrafast lasers and indicates an exponential growth in last two decades. The figure discloses an interesting trend: the remarkable development of ultrafast Er-fiber lasers—spurred by optical communication booming in 1990s—was eventually overtaken by ultrafast Yb-fiber lasers in 2009; in the same year, ultrafast Tm-fiber/Ho-fiber lasers started to advance at a rapid pace.

The advances in ultrafast fiber lasers have been well documented in the last decade by many excellent review papers (Brida et al., 2014, Fermann and Hartl, 2009, Fermann and Hartl, 2013, Galvanauskas, 2001, Jackson, 2012, Jauregui et al., 2013, Limpert et al., 2006, Limpert et al., 2007, Limpert et al., 2011, Limpert et al., 2014, Nilsson and Payne, 2011, Richardson et al., 2010, Shi et al., 2014, Tunnermann et al., 2005, Tunnermann et al., 2010, Xu and Wise, 2013, Zervas, 2014, Zervas and Codemard, 2014, Nilsson et al., 2003), most of which focus on energy/power scalability of ultrafast Yb-fiber lasers. Indeed, laser parameters (e.g., repetition rate, spectral bandwidth, pulse duration, pulse energy, wavelength tuning range, average power) define the performance of an ultrafast laser. The last decade has witnessed a continuous expansion of the parameter space in which ultrafast fiber lasers can operate. In this paper, we review the enabling technologies that has continued to push the boundaries of the laser parameters. This review is structured as follows. In the next section, we present a brief introduction to nonlinear fiber optics that deals with propagation of femtosecond pulses inside optical fibers followed by a discussion of ultrafast fiber oscillators/amplifiers. We then continue to review the last-decade progress in enlarging the coverage of laser parameters—such as repetition rate, pulse energy, average power, and center wavelength—of ultrafast fiber laser systems. The last section presents conclusion and outlook.

## Nonlinear Phenomena Associated with Propagation of Ultrashort Pulses in Active and Passive Fibers

An ultrafast fiber laser system undoubtedly involves propagation of ultrashort pulses inside passive fibers and active fibers. Owing to long interaction length, tight confinement of light inside fiber core area, and high peak power from an ultrashort pulse, such propagation gives rise to various nonlinear phenomena. Understanding how an ultrafast fiber laser works and then improving its performance highly relies on the knowledge of nonlinear fiber optics, a field that explicitly investigates the nonlinear propagation of ultrashort optical pulses inside fibers (Agrawal, 2006). The propagation can be accurately described by the following generalized nonlinear Schrödinger equation (GNLSE) that takes into account both linear and nonlinear effects:

(Equation 1) $$\frac{\partial A}{\partial z}+\left(\sum_{n\geq 2}\beta_{n}\frac{i^{n-1}}{n!}\frac{\partial^{n}}{\partial T^{n}}\right)A=i\gamma \left(1+\frac{i}{w_{0}}\frac{\partial }{\partial T}\right)\left(A\left(z,T\right)\int_{-\infty }^{+\infty }R\left(t'\right){\left|A\left(z,T-\text{t'}\right)\right|}^{2}dt'\right)+\frac{g}{2}A$$

where A(z,T) describes the slowly varying amplitude envelope of the pulse. ${\text{β}}_{\text{n}}$ represents the n^th^-order fiber group-velocity dispersion (GVD). γ is the nonlinear parameter defined as $\text{γ}={\text{ω}}_{0}n_{2}/\left(cA_{eff}\right)$. ${\text{ω}}_{0}$ is the center frequency, $n_{2}$ the nonlinear-index coefficient of the fiber material, cthe light speed in vacuum, and $A_{eff}$ the mode-field area (MFA). R(t) includes both the instantaneous electronic and delayed molecular responses (i.e., Raman response) and is normally given by:

(Equation 2) $$R\left(t\right)=\left(1-f_{R}\right)\delta \left(t\right)+f_{R}h_{R}\left(t\right)$$

where $f_{R}$ represents the fractional contribution of the Raman response to nonlinear polarization $P_{NL}$. $h_{R}\left(t\right)$ denotes the Raman response function. The last term on the right-hand side of Equation 1 accounts for optical fiber amplification; that is, g > 0 (g = 0) corresponds to pulse propagation inside an active (passive) optical fiber.

As the essential equation in the field of nonlinear fiber optics, GNLSE describes complicated nonlinear pulse evolution (Agrawal, 2006) and can be numerically solved by the split-step Fourier method. Nevertheless, to clarify the physics behind a specific nonlinear phenomenon, reduced forms of GNLSE are usually adopted for analytical analysis. Below is a list of frequently encountered examples in the ultrafast fiber laser technology.

### Soliton Formation

If only the second-order dispersion and self-phase modulation (SPM) are considered, Equation 1 can be simplified to the standard NLSE

(Equation 3) $$\frac{\partial A}{\partial z}+i\frac{\beta_{2}}{2}\frac{\partial^{2}A}{\partial T^{2}}=i\gamma {\left|A\right|}^{2}A$$

For a pulse propagating in a fiber with positive GVD (i.e., *β*_2_ > 0), both SPM and GVD exert positive chirp to the pulse. For enough propagation distance, optical wave breaking occurs that manifests as rapid oscillations appearing near pulses edges (Anderson et al., 1992, Tomlinson et al., 1985). In contrast, negative GVD (i.e., *β*_2_ < 0) allows soliton formation. Owing to a balance between dispersion and nonlinearity, a fundamental soliton pulse maintains its profile during the propagation inside a passive fiber. The pulse energy needs to satisfy the well-known soliton area theorem:

(Equation 4) $$E=\frac{1}{\gamma }\frac{\left|\beta_{2}\right|}{T_{0}^{2}}=\frac{cA_{eff}\left|\beta_{2}\right|}{\omega_{0}n_{2}T_{0}^{2}}$$

where $T_{0}$ is connected to the full-width-at-half-maximum (FWHM) of the pulse by $T_{0}\;\sim \;T_{\text{FWHM}}/1.763$ (Agrawal, 2006). Equation 3 accommodates higher-order solitons as well. Unlike a fundamental soliton, higher-order solitons evolve periodically in both the temporal domain and the spectral domain during the propagation. Soliton pulses are stable if only SPM and negative GVD exist. However, as they propagate inside an optical fiber, other effects as included in the GNLSE are inevitable and, under certain circumstances, may be treated as perturbation sources to a soliton. In the following two sections, we briefly discuss two important phenomena related with soliton perturbation.

### Dispersive Wave Generation

If the optical pulse has a broad optical spectrum or its center wavelength is close to the zero-dispersion wavelength of the fiber, higher-order dispersion terms need to be included:

(Equation 5) $$\frac{\partial A}{\partial z}+\left(\sum_{n\geq 2}\beta_{n}\frac{i^{n-1}}{n!}\frac{\partial^{n}}{\partial T^{n}}\right)A=i\gamma {\left|A\right|}^{2}A$$

These higher-order dispersions perturb a fundamental soliton and cause radiation of an optical pulse centered at a new frequency given by the following phase-matching condition:

(Equation 6) $$\sum_{n\geq 2}\frac{{\left(\omega -\omega_{0}\right)}^{n}}{n!}\beta_{n}\left(\omega_{0}\right)-\frac{\gamma p_{0}}{2}=0$$

This phenomenon—widely known as dispersive wave generation (or non-solitonic radiation, fiber-optic Cherenkov radiation)—was first theoretically studied in 1986 (Wai et al., 1986). With the advent of photonic-crystal fibers (PCFs) that allow one to flexibly engineer fiber dispersion, dispersive wave generation attracted intensive research attention under the context of supercontinuum generation (Austin et al., 2006, Cristiani et al., 2004) and later became a useful method for nonlinear wavelength conversion (Chang et al., 2010, Chang et al., 2011). Detailed analysis shows that the sign of the third-order dispersion (TOD, i.e., *β*_3_) determines whether the center wavelength of the dispersive wave pulse is downshifted or upshifted with respect to the soliton center wavelength (Akhmediev and Karlsson, 1995, Karpman, 1993). Positive (negative) TOD produces dispersive wave centered at a shorter (longer) wavelength compared with the soliton.

### Soliton Self-Frequency Shift

In Equation 3, only the instantaneous electronic response of fused silica is included, which gives rise to the SPM effect. Indeed, delayed molecular responses (i.e., Raman response)—represented by the second term in Equation 2—leads to intra-pulse Raman scattering such that the center wavelength of a soliton continuously shifts toward longer wavelength. This phenomenon is known as soliton self-frequency shift (SSFS) (Gordon, 1986, Mitschke and Mollenauer, 1986). Similar as dispersive wave generation, SSFS has been fully explored for investigating supercontinuum generation in PCFs and governs the extension of the spectrum toward the longer wavelength side (Husakou and Herrmann, 2001). In the time domain, SSFS generates wavelength-tunable transform-limited pulses (known as Raman soliton pulses) and the amount of wavelength shift can be readily adjusted by varying the input pulse energy. Together with the rapid development of fiber technology, SSFS constitutes a powerful method to produce wavelength-tunable femtosecond pulses.

### Parabolic Similariton Asymptotically Developed in Fiber Amplifier

As an optical pulse propagates inside an active fiber (e.g., Yb-doped fiber amplifier), Equation 3 should be modified to take into account the gain effect, leading to the following equation:

(Equation 7) $$\frac{\partial A}{\partial z}+i\frac{\beta_{2}}{2}\frac{\partial^{2}A}{\partial T^{2}}=i\gamma {\left|A\right|}^{2}A+\frac{g}{2}A$$

If the fiber exhibits positive GVD (*β*_2_ > 0), the interplay among dispersion, SPM, and gain renders an input pulse of arbitrary shape evolving asymptotically into an amplified, linearly chirped pulse with a parabolic intensity profile (Boscolo et al., 2002, Kruglov et al., 2000, Kruglov et al., 2002). During further propagation, this parabolic pulse evolves in a self-similar manner such that the temporal profile and the chirp rate remain unchanged while the pulse duration, peak power, and spectral bandwidth increase exponentially with the distance. Such an optical pulse is referred to as similariton or, more specifically, parabolic similariton. First experimentally observed in an Yb-fiber amplifier (Fermann et al., 2000), parabolic similariton was soon found in Raman fiber amplifier (Finot et al., 2003), fiber oscillators (Ilday et al., 2004, Oktem et al., 2010), and dispersion-decreasing fibers (Finot et al., 2007, Hirooka and Nakazawa, 2004). Indeed, similariton is a universal phenomenon and can emerge from optical beam propagation (parabolic spatial similariton) (Chang et al., 2006) or an incoherent nonlinear system (incoherent similariton) (Chang et al., 2005). Detailed progress in research on similariton was well documented in review papers (Chong et al., 2015, Dudley et al., 2007, Finot et al., 2009).

## Main Building Blocks of Ultrafast Fiber Laser Systems

Most ultrafast fiber lasers in practical use are configured in a master-oscillator-power-amplifier (MOPA) architecture; that is, an ultrafast fiber oscillator provides stable pulses, which are then amplified by subsequent fiber amplifiers to boost the average power and pulse energy. In the following we briefly discuss ultrafast fiber oscillator and amplifier, respectively.

### Ultrafast Fiber Oscillator

In a MOPA system, the fiber oscillator is passively mode-locked at the fundamental repetition rate. Mode-locking for producing femtosecond pulses is achieved through saturable absorption of the intra-cavity circulating pulse. Such saturable absorption may be implemented by a non-fiber device, known as saturable absorber, which involves direct material absorption. During the past decade, many ultrafast fiber oscillators—especially Er-fiber oscillators—were mode-locked by a saturable absorber made of novel materials, such as carbon nanotube (Set et al., 2004), graphene (Bao et al., 2009), perovskite (Hong et al., 2018), transition metal dichalcogenides (Woodward and Kelleher, 2015), and topological materials (Liu et al., 2016b), to name a few. Nevertheless, semiconductor saturable absorber mirrors (SESAM)—an old technology dated to 1990—are widely believed to still outperform the above-mentioned devices (Keller et al., 1990, Keller et al., 1996, Okhotnikov et al., 2004).

**Figure 2:**
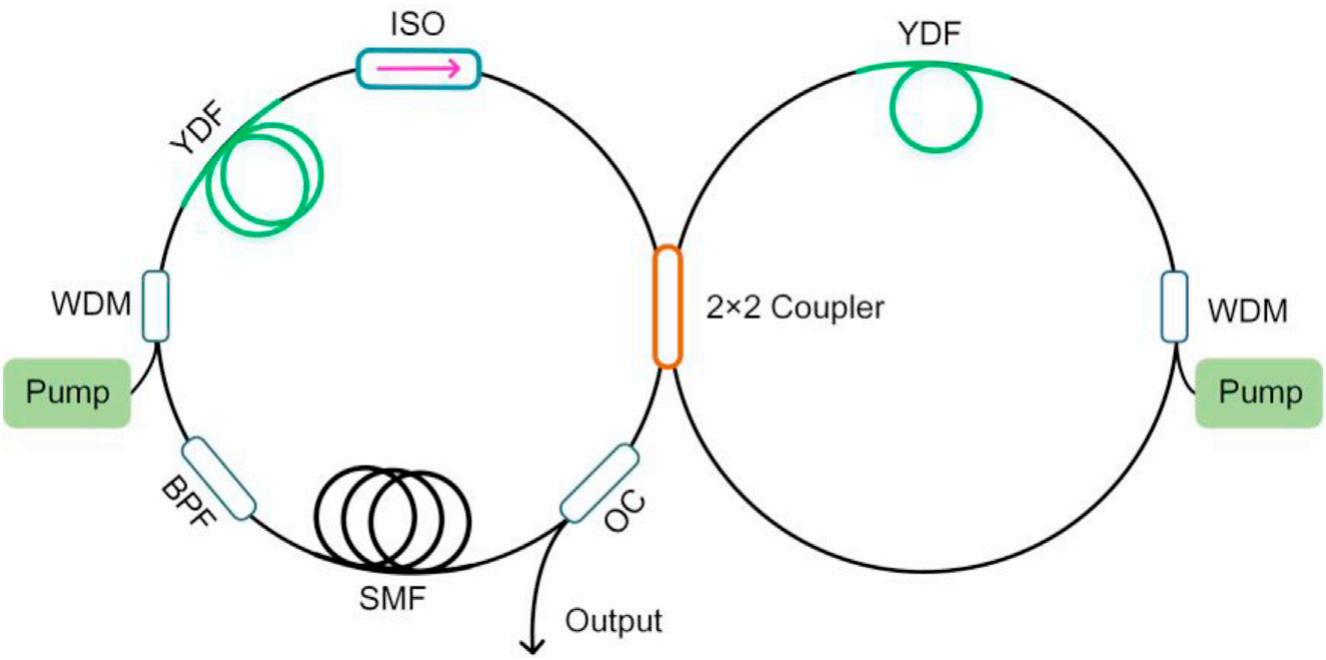
Experimental Setup of a Mode-Locked Yb-Doped All-PM-Fiber Oscillator Mode-Locked by NALM The left loop constitutes the laser main cavity and the right one is an NALM. YDF, Yb-doped fiber; WDM, wavelength division multiplexer; ISO, isolator; BPF, bandpass filter; OC, output coupler.

Besides saturable absorbers made from real materials that involve light absorption, an alternative is to employ fiber-optic nonlinear effects followed by a device to achieve effective saturable absorption. For example, propagation of an elliptically polarized pulse inside an optical fiber may experience nonlinear polarization evolution (NPE); that is, different parts of the pulse (e.g., peak versus wing) exhibit different polarization states. Consequently, a properly aligned polarizer allows more transmission of the peak than the wing and the transmitted pulse becomes shorter. The NPE together with the polarizer thus forms an artificial saturable absorber to mode-lock fiber lasers (Fermann et al., 1993); the resulting lasers are often called NPE fiber lasers. Another type of artificial saturable absorber is configured as a fiber loop connected with the laser cavity by a fiber coupler; typical implementation includes nonlinear-optical loop mirror (Doran and Wood, 1988) and nonlinear amplifying loop mirror (NALM) (Fermann et al., 1990a, Hänsel et al., 2017, Jiang et al., 2016). The intra-cavity pulse is split into two replicas by the coupler before entering the fiber loop such that one replica propagates in the clockwise direction and the other in the counter-clockwise direction. After traveling one round trip in the loop, these two pulses accumulate different nonlinear phase shift and interfere at the coupler before they return to the laser cavity. Figure 2 shows an Yb-doped fiber oscillator constructing from all polarization-maintaining (PM) fibers mode-locked by NALM (Yu et al., 2018b). Two optical loops (main loop on the left and NALM loop on the right) constructed from PM fiber components are connected by a 2 × 2 coupler to form a figure-of-eight cavity. The main loop provides cavity for oscillation, whereas the NALM loop behaves as an artificial saturable absorber that enables mode-locking. This all-fiber oscillator can emit 6-MHz, 93-fs pulses with 10 nJ pulse energy after external compression (Yu et al., 2018b).

Recently, a new type of fiber oscillator, dubbed as Mymeshev oscillator, emerged and quickly attracted intensive research interest (Liu et al., 2017b, Liu et al., 2019, Regelskis et al., 2015, Sidorenko et al., 2018). In a Mymeshev oscillator, fiber-optic nonlinearity that causes substantial spectral broadening and two bandpass optical filters that center at different wavelengths work jointly as two cascaded Mymeshev regenerators (Regelskis et al., 2015). Only a suitable ultrashort pulse can circulate inside the laser cavity; a CW background (or weak pulse) cannot exist because two bandpass filters have no overlap in transmission. These cascaded Mymeshev regenerators are equivalent to an artificial saturable absorber with 100% modulation depth (Liu et al., 2017b).

Passive mode-locking ensures that the intra-cavity pulse can circulate stably in the fiber oscillator cavity. However, the evolution of the intra-cavity pulse in one round trip highly depends on the cavity dispersion map. In other words, managing the cavity dispersion can control the operation state of a fiber oscillator. Depending on the amount of net cavity dispersion and its sign, the circulating pulse inside the cavity ranges from sub-ps to >10 ps in duration corresponding to different mode-locking regimes (e.g., soliton, stretched pulse, similariton, and dissipative soliton) (Chong et al., 2008, Wise et al., 2008). For Yb-fiber laser at ~1.03 μm, conventional fibers exhibit positive GVD. To manage the cavity dispersion, negative GVD is introduced by grating pairs (Treacy, 1969), PCFs (Birks et al., 1999), or chirped fiber Bragg gratings (CFBGs) (Hill et al., 1994). For Er-fiber laser at ~1.55 μm, many types of solid-core fibers—such as dispersion-shifted fibers, dispersion compensation fibers, dispersion decreasing fibers—are developed to engineer the fiber dispersion. At this wavelength, both passive and active PM fibers with either positive or negative GVD are commercially available.

### Ultrafast Fiber Amplifier

Although much research effort has been devoted in power/energy scaling a fiber oscillator, a conventional wisdom toward producing high-power/energy femtosecond pulses is to rely on a MOPA system. In a MOPA system, the master oscillator only needs to provide low-power/energy pulses and thus can be carefully engineered to achieve low noise, high compactness, and extreme robustness. The weak seeding pulses are then amplified in subsequent fiber amplifiers by orders of magnitude in pulse energy or average power. In most cases, the performance of a fiber laser system strongly depends on the amount of nonlinear phase shift accumulated by the pulse. For a fixed nonlinear phase shift, increasing the MFA allows higher pulse peak-power inside the gain fiber. To date, most fiber amplifiers that output >1 W average power are constructed from double-clad large-mode-area (LMA) fibers. The double-clad structure—first demonstrated in 1988—has become the standard technique that allows pumping an active fiber using high-power multimode laser diodes (Snitzer et al., 1988). In the last two decades, many groups proposed and demonstrated double-clad active fibers with increased core MFA, such as chirally coupled core fibers (Liu et al., 2007), leakage channel fibers (Dong et al., 2009), and rod-type fibers (Limpert et al., 2005). Recently developed rod-type large-pitch fibers (LPFs) delocalize the higher-order modes resulting in their poor overlapping with the doped core region; as a result, the differential gain between the fundamental mode and higher-order modes guarantees robust single-mode operation of amplifiers constructed from rod-type LPF amplifiers (Stutzki et al., 2011). The mode-field diameter (MFD) of an Yb-doped rod-type LPF can exceed 100 μm, corresponding to an MFA 100 times larger than that offered by conventional single-mode fibers (Limpert et al., 2012).

## Enlarge the Parameter Space to Expand Fiber Laser Toolbox

An ultrafast fiber laser system is well characterized by several measurable parameters such as repetition rate, pulse energy, pulse duration, peak power, average power, and center wavelength. In practice, a specific application demands optimization of a subset of these parameters. Indeed, optimizing some parameters unavoidably compromises other parameters in reality owing to technical limitations. In this section we review the typical parameter space of ultrafast fiber laser systems.

### Repetition Rate

Scientific and industrial applications may demand femtosecond pulses with the repetition rate ranging from 1 kHz to >10 GHz. The cavity length determines the repetition rate of a fundamentally mode-locked ultrafast fiber oscillator. Most fiber oscillators include several-meter-long optical fibers and the typical repetition rate is tens of MHz. Adding more passive fibers into the cavity can lower down the repetition rate. However, reducing the repetition rate below 1 MHz from a ring-cavity oscillator corresponds to a cavity of about 200 m in length. Such a long cavity suffers from two drawbacks: (1) the oscillator is vulnerable to ambient disturbances, which may destroy mode-locking and (2) the output pulse develops nonlinear and gigantic chirp making the pulse hard to compress. In practical implementation, ultrashort pulses with a repetition rate below 10 MHz are derived from an ultrafast fiber laser system via pulse picking enabled by an acousto-optic pulse picker.

Mode-locking of a fiber laser at a repetition rate above 200 MHz is challenging as well. Especially as the repetition rate exceeds 1 GHz, the cavity fiber is less than 10 cm in length and therefore the active fiber must be highly doped to provide enough gain. Table 1 lists representative ultrafast Yb-doped and Er-doped fiber oscillators fundamentally mode-locked with the repetition rate beyond 150 MHz (Byun et al., 2010, Chen et al., 2007, Chen et al., 2012b, Cheng et al., 2017, Cheng et al., 2018, Du et al., 2017, Huang et al., 2017, Ilday et al., 2005, Li et al., 2013, Li et al., 2014a, Li et al., 2014b, Li et al., 2015, Liu et al., 2018, Ma et al., 2010, Martinez and Yamashita, 2011, Martinez and Yamashita, 2012, Song et al., 2019a, Song et al., 2019b, Wang et al., 2011, Wang et al., 2019, Wu et al., 2015a, Xing et al., 2015, Yang et al., 2012, Zhang et al., 2016).

**Table 1:** The Evolution of High-Repetition-Rate Ultrafast Er/Yb Fiber Laser

	Cavity	λ (nm)	E_p_ (pJ)	τ_p_ (fs)	f_rep_ (GHz)	Reference
YDF	Linear (SA)	1,025	17.7	206	3	(Chen et al., 2012b)
		1,059	8–16	2,600	5	(Cheng et al., 2017)
		1,041	19	3,400	3	(Cheng et al., 2018)
		1,060	NA	3,200	3.1	(Wang et al., 2019)
		1,050	NA	3,200	7.0	(Wang et al., 2019)
		1,047	NA	1,900	12.5	(Wang et al., 2019)
	Ring (NPE)	1,035	150	70∗	0.162	(Ilday et al., 2005)
		1,030	320	156	0.503	(Wang et al., 2011)
		1,030	920	502	0.605	(Yang et al., 2012)
		1,035	280	68∗	0.75	(Li et al., 2013)
		1,020	731	98∗	0.616	(Li et al., 2014a)
		1,030	600	64∗	1.0	(Li et al., 2015)
		1,027	214	215	0.7	(Liu et al., 2018)
EDF	Linear (SA)	1,573	283	187	0.967	(Byun et al., 2010)
		1,600	0.15	680	4.24	(Martinez and Yamashita, 2011)
		1,560	0.26	940	9.64	(Martinez and Yamashita, 2011)
		1,563	0.32	790	19.45	(Martinez and Yamashita, 2011)
		1,562	0.16	865	9.67	(Martinez and Yamashita, 2012)
		1,556	13	935	0.463	(Wu et al., 2015a)
		1,558	7.3	379	1.0	(Huang et al., 2017)
		1,588	NA	639	3.2	(Cheng et al., 2018)
		1,558	160.5	244∗	2.68	(Song et al., 2019b)
		1,553	5.34	550	1.03	(Song et al., 2019a)
	Ring (NPE)	1,565	154.6	167	0.194	(Chen et al., 2007)
		1,555	310	37.4∗	0.225	(Ma et al., 2010)
		1,553	420	56.5	0.202	(Xing et al., 2015)
		1,560	174.1	180	0.517	(Zhang et al., 2016)
		1,561	39	115	0.354	(Du et al., 2017)
	NPE + SA	1,552	310	41.9∗	0.212	(Li et al., 2014b)

YDF, Yb-doped fiber; EDF, Er-doped fiber; SA, saturable absorber; NPE, nonlinear polarization evolution. Pulse duration marked with ∗ indicates that the pulses are externally compressed or the duration corresponds to calculated transform-limited pulse.

**Figure 3:**
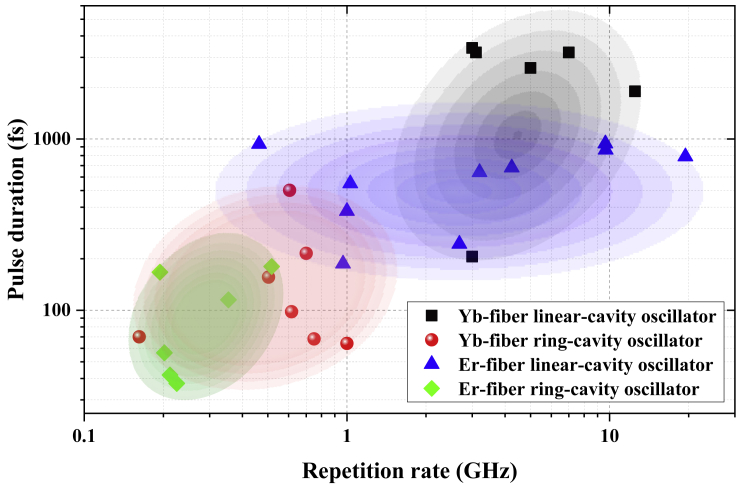
Pulse Duration as a Function of Repetition Rate for Ultrafast Yb-Fiber and Er-Fiber Oscillators with the Repetition Rate Exceeding 150 MHz The results are grouped into four categories: (1) Yb-fiber linear-cavity oscillators (black squares), (2) Yb-fiber ring-cavity oscillators (red circles), (3) Er-fiber linear cavity oscillators (blue triangles), and (4) Er-fiber ring-cavity oscillator (green diamonds).

Limited by the available power from single-mode pump diodes, increased repetition rate is usually accompanied by reduced pulse energy. For GHz ultrafast fiber lasers, the pulse energy may be as low as sub-pJ (Martinez and Yamashita, 2011, Martinez and Yamashita, 2012). Because of reduced pulse energy and short fiber length, the intra-cavity pulse accumulates less nonlinear phase shift in each round trip and the associated nonlinear pulse shaping is weakened, leading to narrower optical spectrum and longer pulse. Figure 3 illustrates the pulse duration as a function of repetition rate for ultrafast Yb-doped and Er-doped fiber lasers; each type of fiber lasers can be configured in linear cavity or ring cavity. In general, given the same type of active fibers (Yb-doped versus Er-doped), linear-cavity configuration results in higher repetition rate than can be achieved from ring-cavity configuration.

It is noteworthy that highly doped Yb-fibers exhibit positive GVD, which necessitates dispersion compensation for the oscillator cavity in order to produce femtosecond pulses. For example, we demonstrated a 3-GHz fiber oscillator using 1-cm, heavily Yb-doped phosphate glass fiber as the gain medium (Chen et al., 2012b). To compensate for the positive GVD, the output coupler was specially designed with custom coating structures that provides −1,300 fs^2^ group-delay dispersion at 1.03 μm making the net cavity dispersion slightly negative. Such a compact Yb-fiber oscillator produces 3-GHz pulses with the pulse duration as short as 206 fs (Chen et al., 2012b). Recently, Yb-fiber oscillators with >5-GHz repetition rate are demonstrated (Cheng et al., 2017, Cheng et al., 2018, Liu et al., 2018, Wang et al., 2019); without dispersion compensation, these oscillators emit picosecond pulses. In contrast, highly doped Er-fibers have negative GVD and the resulting oscillators can operate at soliton mode-locking regime without requirement of dispersion compensation. To date, the highest repetition rate of a fundamentally mode-locked Er-fiber oscillator is 19.45 GHz and the output pulse has a duration of 790 fs (Martinez and Yamashita, 2011).

Some applications (e.g., all-optical sampling, precision metrology) desire that the repetition rate of an ultrafast laser can be tuned in a range exceeding ±1%. In the context of frequency comb applications, the repetition rate of a fiber oscillator determines the frequency spacing between two adjacent comb lines. In this scenario, tuning the repetition rate can precisely vary the comb spacing. Since the repetition rate is determined by the round-trip time of the intra-cavity circulating pulse, tuning the repetition rate was normally achieved by introducing a tunable optical delay line into the cavity of a passively mode-locked fiber oscillator (Hundertmark et al., 2004, Liu et al., 2010, Washburn et al., 2004, Wu et al., 2015b, Yang et al., 2016).

In recent years, dual-comb spectroscopy has emerged as a powerful spectroscopic technology, which holds promise for many important applications (Coddington et al., 2016, Ideguchi et al., 2014, Millot et al., 2016, Suh et al., 2016, Villares et al., 2014, Yu et al., 2018a). This technology requires two frequency combs with different comb spacing, which are normally achieved based on two ultrafast oscillators mode-locked at different repetition rates. A more attractive way to implement such a dual comb is to mode-lock a single fiber oscillator in a way such that it emits two pulse trains at different repetition rates (Mehravar et al., 2016, Zhao et al., 2016). As a result, the resulting two pulse trains can maintain mutual coherence. This type of ultrafast fiber oscillators are carefully designed such that two pulses circulate inside the oscillator cavity. To guarantee different round-trip times (and therefore different repetition rates), special efforts should be undertaken to ensure that these two intra-cavity pulses are different in polarization (Akosman and Sander, 2017, Deng et al., 2019, Guo et al., 2019, Nakajima et al., 2019), center wavelength (Chen et al., 2019, Guo et al., 2018, Hu et al., 2017, Hu et al., 2018, Jin et al., 2020, Li et al., 2018b, Liao et al., 2018, Mehravar et al., 2016, Nitta et al., 2018, Olson et al., 2018, Pawliszewska et al., 2020, Shi et al., 2018, Wang et al., 2016, Yun et al., 2012, Zhao et al., 2011, Zhao et al., 2012a, Zhao et al., 2016, Zhao et al., 2019), or propagation path (Cui and Liu, 2013, Kayes et al., 2018, Mao et al., 2013). Table 2 summarizes the typical results of these dual-comb mode-locked fiber oscillators in terms of laser type, center wavelengths of the two pulse trains, repetition rate, and repetition-rate difference.

**Table 2:** Dual-Comb Fiber Oscillators that Emit Two Pulse Trains at Different Repetition Rates

	Cavity	$\lambda_{1}$ (nm)	$\lambda_{2}$ (nm)	$f_{\mathrm{rep}}$ (MHz)	Δf (kHz)	Reference
EDF	Ring	1,532.2	1,557.3	9.09	0.58	(Zhao et al., 2011)
		1,532.4	1,556	15.75	0.466	(Zhao et al., 2012a)
		1,550	1,562	7.68	889	(Mao et al., 2013)
		1,560	1,560	7.05	4,480	(Cui and Liu, 2013)
		1,533	1,544	52.74	1.25	(Zhao et al., 2016)
		1,554.3	1,555.1	72.38	0.082	(Mehravar et al., 2016)
		1,531.4	1,556.1	32.07	1.63	(Hu et al., 2017)
		1,531.7	1,555.2	50.64	2.13	(Shi et al., 2018)
		1,533	1,543	64.55	0.248	(Hu et al., 2018)
		1,530	1,560	48.80	0.217	(Nitta et al., 2018)
		1,559.4	1,559.6	20.9	0.009	(Guo et al., 2018)
		1,570	1,581	40.52	0.93	(Li et al., 2018b)
		1,554.5	1,565.3	24.83	0.633	(Zhao et al., 2019)
		1,560	1,560	98.94	1.4	(Guo et al., 2019)
		1,551.9	1,560	68.67	2.621	(Chen et al., 2019)
		1,539.5	1,564.4	21.20	0.009	(Nakajima et al., 2019)
		1,530	1,556	28.90	0.058	(Jin et al., 2020)
	Figure-8	1,572	1,587	3.28	NA	(Yun et al., 2012)
	Linear	1,541	1,541	46.73	11.989	(Deng et al., 2019)
TDF	Ring	1,943.6	1,972	23.48	2.633	(Wang et al., 2016)
		1,956.8	1,979.2	10.77	0.316	(Wang et al., 2016)
		1,911	1,913	16.57	1.3	(Kayes et al., 2018)
		1,917	1,981	71.88	3.27	(Liao et al., 2018)
		1,870^a^	1,870^a^	60.78	0.229^a^	(Olson et al., 2018)
T/HDF	Linear	1,975	1,975	67.62	0.51	(Akosman and Sander, 2017)
HDF	Ring	2,021	2,096	48.29^a^	8^a^	(Pawliszewska et al., 2020)

EDF, Er-doped fiber; TDF, Tm-doped fiber; T/HDF, Tm/Ho-doped fiber; HDF, Ho-doped fiber.

a Minimum value.

### Energy and Power Scaling

A fiber oscillator normally emits ultrashort pulses with nJ-level pulse energy, and the average power is below 100 mW. Further energy/power scaling relies on a MOPA system that involves amplifying ultrashort pulses in active fibers. The first ultrafast fiber amplifier—which was reported in 1989—was constructed from an erbium-doped fiber pumped by a diode laser (Nakazawa et al., 1989, Suzuki et al., 1989). Compared with Er-fiber MOPA system, Yb-fiber amplifiers feature much higher optical-to-optical conversion efficiency (~80%) and hence become the best fiber laser system capable of delivering high-energy and high-power femtosecond pulses (Paschotta et al., 1997). Amplification of ultrashort pulses has to deal with fiber-optic nonlinearities because excessive nonlinear phase shift may introduce complicated chirp to the amplified pulse, making it uncompressible. We divide amplification techniques into two main categories—linear amplification and nonlinear amplification—depending on whether the optical spectrum of the amplified pulses becomes much broader than the input spectrum.

#### Linear Amplification

**Figure 4:**
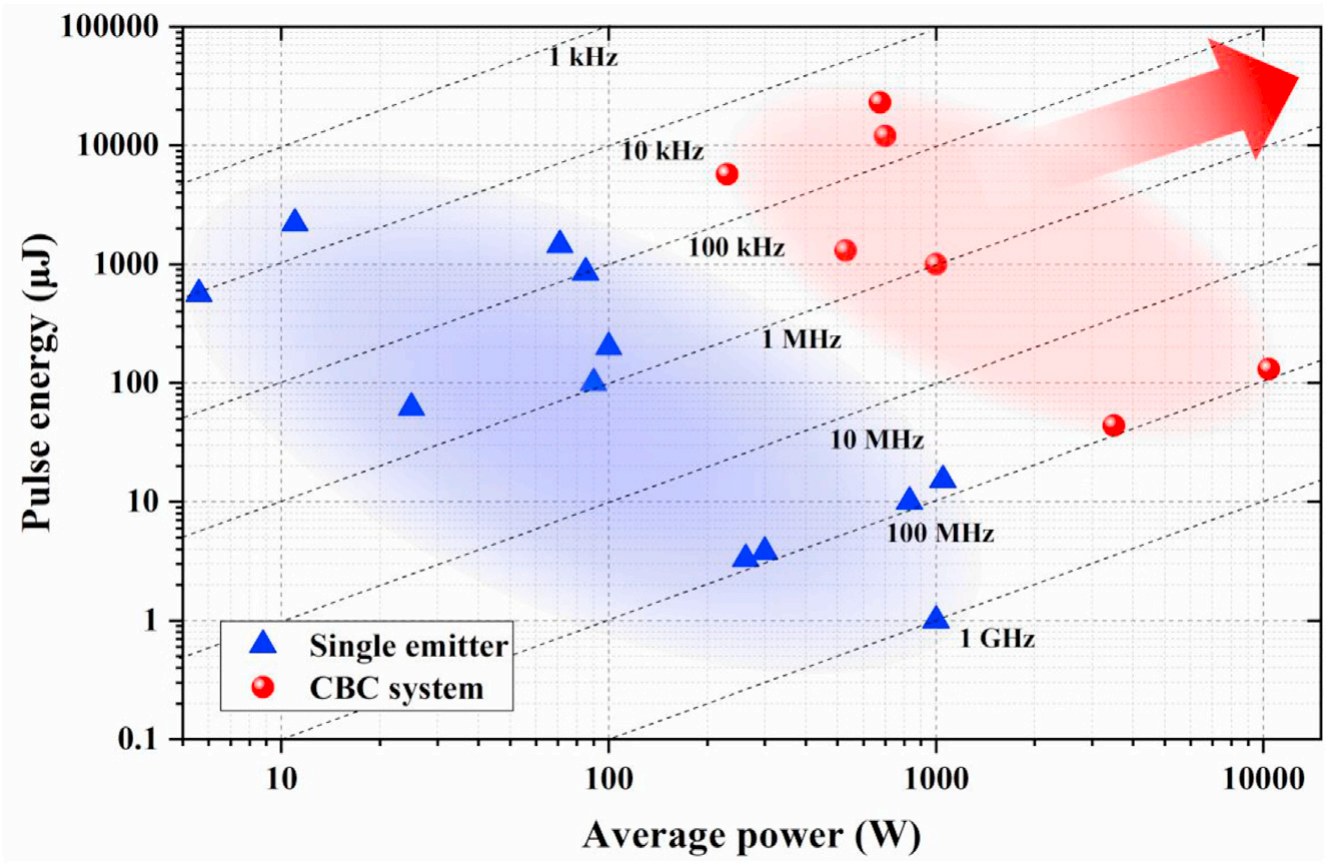
Pulse Energy as a Function of Average Power for Yb-Fiber CPA Systems Triangles: single emitters; circles: CPA systems incorporating DPA and CBC. Dashed lines mark the repetition rate.

Chirped-pulse amplification (CPA)—the mature technique used in solid-state amplifiers—is a linear amplification technique, which was introduced in fiber amplifiers in 1993 (Stock et al., 1993) and soon adopted by most fiber amplifiers that produced pulses with >1 μJ pulse energy. A fiber CPA system typically includes (1) an ultrafast fiber oscillator providing weak seeding pulse, (2) a pulse stretcher that elongates the seeding pulse up to nanosecond level, (3) one- or multi-fiber amplifiers, and (4) a pulse compressor that dechirps the amplified pulse back to nearly transform-limited pulse duration. In the early demonstrations, Martinez-type diffraction-grating pair (Martinez, 1987) and Treacy-type grating pair (Treacy, 1969) are used as the stretcher and compressor, respectively. These grating pairs feature a large footprint and need precise alignment. These drawbacks are avoided by replacing diffraction gratings with chirped volume Bragg gratings (CVBGs) constructed from photo-thermo-refractive glass (Chang et al., 2009, Liao et al., 2007). Although CVBGs are compact, they are free-space devices as well. To achieve a MOPA of all-fiber format, the stretcher is usually constructed from PM fibers or CFBGs that provide negative group-delay dispersion. To accommodate the amplified pulses with large pulse energy, hollow-core photonic-bandgap fibers were used as the compressor (de Matos et al., 2003, Limpert et al., 2004). Although air exhibits much smaller nonlinear refraction coefficient than fused silica, compression of μJ-level pulses encounters onset of nonlinear effects, which degrades the quality of compressed pulses. To date, nearly all the fiber CPA systems with >1 μJ pule energy employ Treacy-type grating pair as the compressor (Bonod and Neauport, 2016). In a fiber CPA system, special attention should be paid to the precise compensation of TOD in order to ensure high-quality pulse compression. Fortunately, several groups demonstrated that intentionally accumulating a certain amount of nonlinear phase shift can mitigate the effect of TOD and improve the compressed-pulse quality (Kalaycioglu et al., 2010, Kuznetsova and Wise, 2007, Shah et al., 2005, Zhou et al., 2005). Thanks to the rapid development of double-clad Yb-doped LMA fibers, femtosecond pulses centered at ~1.03 μm with >100 μJ pulse energy can be routinely obtained in an Yb-fiber CPA system. The blue triangles in Figure 4 show the representative Yb-fiber CPA systems (Röser et al., 2007, Eidam et al., 2010, Eidam et al., 2011, Hao et al., 2009, Kim et al., 2015, Plotner et al., 2017, Roser et al., 2007, Wan et al., 2013, Yu et al., 2016, Zhao et al., 2012b, Zhao and Kobayashi, 2016, Ogino et al., 2013). In a practical fiber CPA system, the seeding pulse is stretched at most to a duration of ~2 ns. To avoid the detrimental effect of self-focusing, the peak power of the amplified pulse in a fiber amplifier needs to remain well below the catastrophic threshold power (e.g., 4 MW for linearly polarized pulse amplified in Yb-fiber amplifier). These two factors (i.e., stretched-pulse duration and self-focusing) limit the amplified pulse energy; among reported results, the highest pulse energy obtained from a single Yb-fiber CPA system (“single emitter”) is about 2.2 mJ (Eidam et al., 2011).

In addition to pulse energy, average power constitutes another important parameter. Fiber laser systems that can deliver femtosecond pulses featuring both high energy (>1 mJ) and high power (>1 kW) are highly desired by high-field science. Scaling of average power in fiber amplifiers is eventually prevented by the onset of thermal modal instability (Dong, 2013, Jauregui et al., 2012, Smith and Smith, 2011, Ward et al., 2012), which limits the average power to 1 kW for Yb-fiber amplifiers that deliver diffraction-limited beams. To further scaling pulse energy and average power, divided pulse amplification (DPA) (Zhou et al., 2007) and coherent beam combining (CBC) are introduced into fiber CPA systems. A thorough review of this technique can be found in Hanna et al., 2016, Klenke et al., 2018. The red circles in Figure 4 present recent Yb-fiber CPA systems incorporating DPA and CBC (Kienel et al., 2016, Klenke et al., 2013, Klenke et al., 2014, Mueller et al., 2020, Muller et al., 2016, Muller et al., 2018, Stark et al., 2019); femtosecond pulses with >100 mJ energy and >10 kW average power are expected in the near future.

One drawback associated with the fiber CPA system lies in the fact that the compressed-pulse duration is typically ≥200 fs limited by gain narrowing and residual dispersion mismatch. Many important applications especially high-field science demand much shorter pulse duration. To meet such duration requirement, subsequent nonlinear pulse compression becomes necessary at the expense of system complexity and throughput efficiency. Depending on the pulse energy offered by a fiber CPA system, the choice of proper nonlinear media can be solid-core LMA fibers (Eidam et al., 2008, Jocher et al., 2012), hollow-core Kagome PCFs (Debord et al., 2014, Guichard et al., 2015), noble gas-filled glass capillaries with sub-mm MFD (Hadrich et al., 2013, Lavenu et al., 2017), and gas-filled multi-pass cell (Lavenu et al., 2019).

#### Nonlinear Amplification

Some applications, for example, cavity-enhanced high-harmonic generation, demand μJ-level pulses with <100 fs pulse duration and tens-of-MHz repetition rate (Cingöz et al., 2012, Gohle et al., 2005, Jones et al., 2005). Such a parameter combination can be achieved via nonlinear amplification; that is, the amplified pulse is transform limited or slightly stretched in duration (normally <1 ps) and accumulates enough nonlinear phase shift during amplification resulting in significantly broadened spectrum to overcome the gain narrowing effect. At the output, the amplified and spectrally broadened pulse is compressed to a duration much shorter than the seeding pulse.

**Figure 5:**
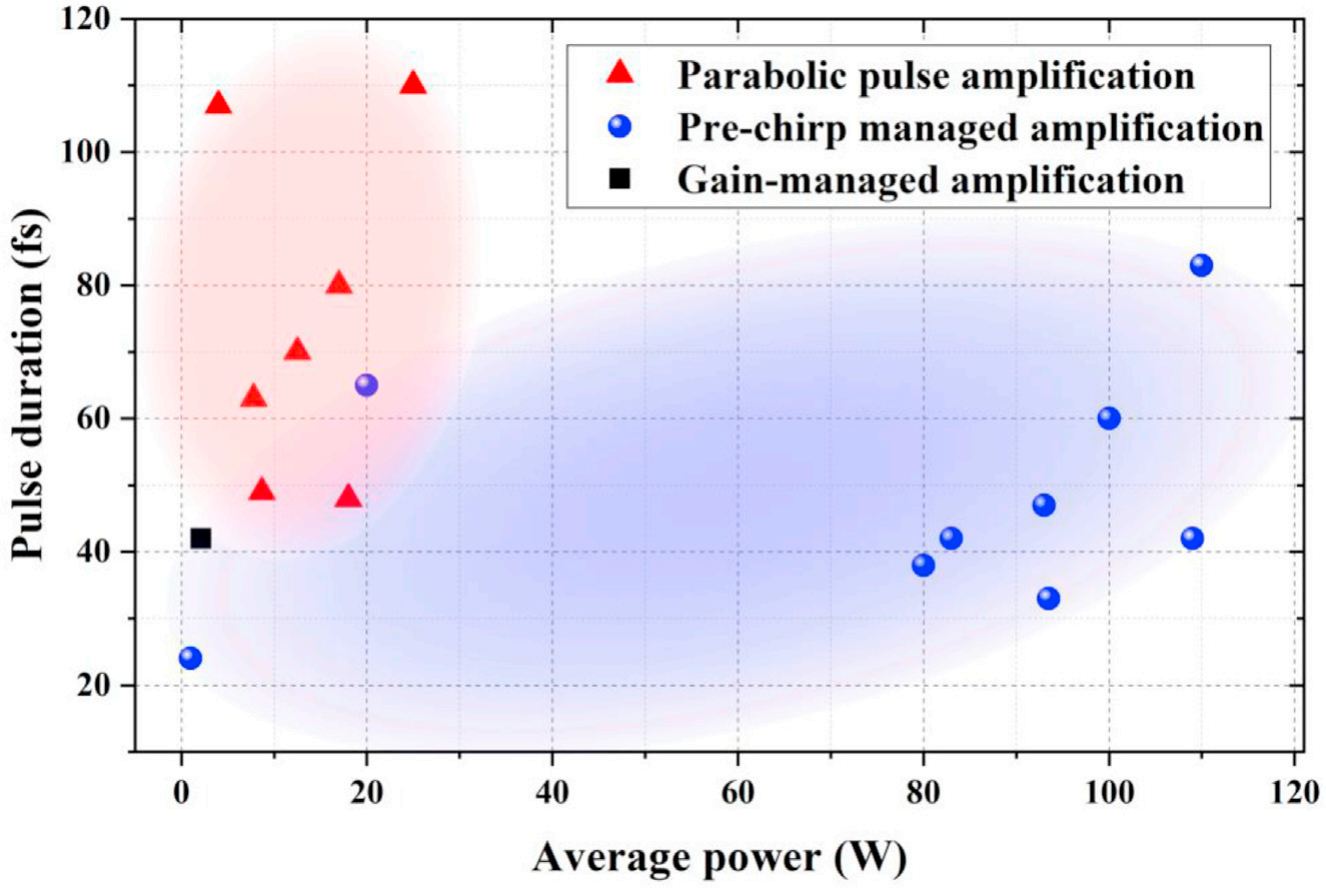
Pulse Duration as a Function of Average Power for Yb-Fiber Amplifiers Based on Parabolic Pulse Amplification (Red Triangles), PCMA (Blue Circles), and Gain-Managed Amplification (Black Square).

Indeed, before the invention of CPA (Strickland and Mourou, 1985), pulse amplification without a stretcher was commonly used. Nonlinear amplification was proposed in 1974, which theoretically demonstrated that, after being amplified in a 2-m-long Nd:glass chain, the initial 1-ns pulse can be compressed down to 125 ps due to SPM-caused spectral broadening in the gain medium (Fisher and Bischel, 1974). However, in reality, the short length (<1 cm) of a solid-state gain medium and the absence of waveguide effect in a bulk material give rise to minimal nonlinear phase shift and spectral broadening. This direct pulse amplification technique unsurprisingly died out in the competition with CPA for solid-state amplifiers. After almost 30 years, this technique revived in fiber amplifiers, in which the interplay of positive GVD, SPM, and gain leads to parabolic similariton with a linear chirp, as we show in section “Parabolic Similariton Asymptotically Developed in Fiber Amplifier.” Such amplified and linearly chirped pulse can be readily compressed by a Treacy-type grating pair. Known as parabolic pulse amplification, which was first demonstrated in 1996 (Tamura and Nakazawa, 1996), this technique was not fully explored until the theoretical discovery and experimental verification of the self-similar solution of an amplified NLSE in 2000 (Fermann et al., 2000). Since then, parabolic pulse amplification has been demonstrated in Yb-doped fiber amplifiers (Deng et al., 2009, Limpert et al., 2002, Malinowski et al., 2004, Papadopoulos et al., 2007, Zaouter et al., 2007, Zaouter et al., 2008) as well as Er-doped fiber amplifiers constructed from dispersion shifted fiber (Nicholson et al., 2004, Ozeki et al., 2004, Ozeki et al., 2005). The red triangles in Figure 5 show the representative results from Yb-fiber parabolic-pulse-amplification system. This amplification scheme has produced sub-50-fs pulses with ~20 W average power and ~300 nJ pulse energy (Deng et al., 2009).

Further energy scaling of parabolic pulse amplification is prevented by finite gain bandwidth for a short gain fiber and by the onset of stimulated Raman scattering for a long gain fiber (Chang et al., 2004, Soh et al., 2006a, Soh et al., 2006b). The typical active-fiber length in a parabolic pulse amplifier is several meters such that the initial pulse asymptotically evolves into the self-similar regime. On the other hand, construction of the last-stage power amplifier tends to employ short-length (<1 m) active fiber in order to reduce nonlinearity and thus increase amplified pulse energy. Unable to evolve into a parabolic similariton, the amplified pulse in high-gain short-length fiber amplifiers is spectrally broadened beyond the gain bandwidth and develops nonlinear chirp. As a result, the compressed-pulse quality is compromised. In 2012, we proposed and demonstrated that fine pre-chirping the seeding pulse chirp prior to nonlinear amplification generated high-quality compressed pulse (Chen et al., 2012a). We refer to this new application technique as pre-chirp managed amplification (PCMA) (Chen et al., 2012a). Using an Yb-doped rod-type LPF as the power amplifier, PCMA allows generation of μJ-level ultrashort pulses with ~40 fs pulse duration and >100 W average power. The blue circles in Figure 5 summarize typical Yb-fiber PCMA systems (Huang et al., 2016, Liu et al., 2015a, Luo et al., 2016, Luo et al., 2018, Song et al., 2017, Wang et al., 2016, Zhao et al., 2014, Huang et al., 2019, Wu et al., 2013). Thanks to pre-chirp management, which adds one more degree of freedom, PCMA can generate amplified pulses with broader spectrum compared with parabolic pulse amplification. As a result, PCMA has the potential to deliver μJ-level pulses with the duration as short as few optical cycles. Recently a new nonlinear amplification technique was proposed, in which the spectral broadening was managed by longitudinally evolving gain shaping (Sidorenko et al., 2019); as a proof of principle, 42-fs compressed pulses were obtained (black square in Figure 5).

### Wavelength Tuning

**Figure 6:**
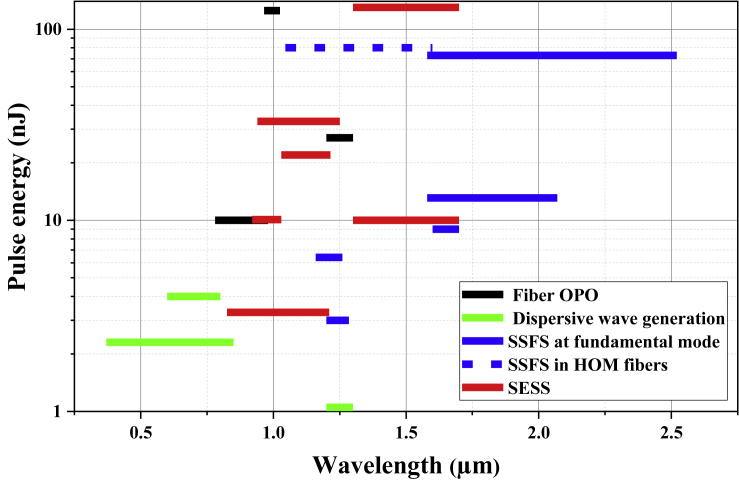
Maximum Pulse Energy versus Wavelength Tuning Range for Different Nonlinear Fiber-optic Methods

It is well known that, among solid-state ultrafast lasers, mode-locked Ti:sapphire laser may be the most successful one largely because the associated huge gain bandwidth permits broadly tuning (>300 nm) the center wavelength of the emitted pulses. Ultrashort pulses with the center wavelength tunable are desired in many microscopy and spectroscopy applications (Lefort, 2017, Taylor, 2016, Tu and Boppart, 2013). Unfortunately, limited by the available gain bandwidth, ultrafast fiber lasers exhibit much narrower tuning range (Ma et al., 2019, Nyushkov et al., 2019). Largely driven by biomedical imaging applications (e.g., multiphoton microscopy), the past decade has witnessed growing research endeavors in developing nonlinear fiber-optic methods to derive wavelength-tunable ultrashort pulses from a source fiber laser. These methods include fiber optical parametric oscillators (OPOs) (Brinkmann et al., 2019, Gottschall et al., 2015, Yang et al., 2018, Zhou et al., 2009), dispersive wave (DW) generation (or so-called Cherenkov radiation) (Chan et al., 2014, Chen et al., 2013, Li et al., 2016, Tauser et al., 2004, Tu et al., 2013, Wang et al., 2015), soliton self-frequency shift (SSFS) (Cadroas et al., 2017, Deng et al., 2015, Fang et al., 2013, Li et al., 2018a, Lim et al., 2014, Rishøj et al., 2019, Takayanagi et al., 2006, van Howe et al., 2007, Yao et al., 2015, Lim et al., 2004), and SPM-enabled spectral selection (SESS) (Chung et al., 2017, Chung et al., 2018, Liu et al., 2016a, Liu et al., 2017a, Niu et al., 2018, Yu et al., 2019). They can significantly expand the wavelength coverage of ultrafast fiber lasers. Table 3 summarizes the representative results including wavelength tuning range, maximum pulse energy, pulse duration, repetition rate, and excitation wavelength. To have a better comparison, Figure 6 plots the maximum pulse energy obtained by nonlinear fiber-optical wavelength conversion as a function of wavelength tuning range. In this figure, we only show the results corresponding to >1 nJ pulse energy.

**Table 3:** Nonlinear Fiber-Optic Methods for Wavelength Conversion

	Tuning Range (μm)	Max. Energy (nJ)	Pulse Duration (fs)	Repetition Rate (MHz)	Excitation Wavelength (μm)
Fiber OPO	1.2–1.3	27	>560	0.785	1.04
	0.78–0.98	10	7,000	40.5	1.04
	1.41–1.54	0.03	10,200	156.2	1.54–1.56
	1.57–1.69	125	350	0.8–20	1.06
Dispersive wave generation	0.96–1.02	0.23	<25	67	1.55
	1.13–1.3	0.13	14	3,000	1.03
	0.75–0.95	2.3	25	50	1.55
	0.37–0.85	>4	>404	54.77	1.03
	0.6–0.8	1.05	125	52.4	1.55
SSFS at fundamental mode	1.2–1.3	0.5	<100	60	1
	1.03–1.33	0.24	100–150	41.3	1.05
	1.05–1.69	73	100	0.66	1.55
	1.58–2.52	6.4	82	50	1.04
	1.16–1.26	0.3	136	3,000	1.03
	1.15–1.35	3	NA	39.6	1.03
	1.2–1.28	9	75	50	1.56
	1.6–1.7	13.1	<100	38.2	1.55
SSFS in HOM fibers	1.58–2.07	0.8	49	80	1.06
	1.05–1.6	80	74	NA	1.04
SESS	1.04–1.6	3.3	70–120	55	1.03
	0.82–1.21	22	50–90	55	1.03
	1.03–1.21	10	50	31	1.55
	1.3–1.7	130	100	31	1.55
	1.3–1.7	10.1	80–117	37	1.03
	0.92–1.03	33	100	1	1.03

Fiber OPOs employ four-wave mixing inside an optical fiber and exhibit certain wavelength tunability (up to ~180 nm). Fiber dispersion gives rise to narrow phase-matching bandwidth, which limits the generated pulse duration and often results in sub-picosecond or picosecond pulses. For example, a fiber OPO pumped by a μJ-level Yb-fiber laser system produces 27-nJ, 560-fs pulses post compression (Gottschall et al., 2015).

Dispersive wave generation as described earlier can generate femtosecond pulses with a duration well below 100 fs. In most cases, the optical fiber exhibits positive TOD and the dispersive-wave pulse has a center wavelength shorter than the excitation pulse. For example, we demonstrated that dispersive-wave pulses centered at 850 nm with 200-nm bandwidth were derived from a 3-GHz Yb-fiber laser system and double-chirped mirrors dechirped these pulses down to 14 fs in duration (Chen et al., 2013). However, as Equation 5 shows, the center wavelength of the generated dispersive wave is mainly determined by the fiber dispersion. Both theoretical and experimental results showed that varying the input pulse peak power can at best tune the center wavelength of the dispersive wave by about 100 nm (Chang et al., 2010, Wang et al., 2015). Increasing the wavelength tuning range can be achieved using several fibers with different zero-dispersion wavelength (Tu et al., 2013). Reference Liu et al. (2015b) presents a thorough review of dispersive wave generation excited by ultrafast fiber lasers.

SSFS can generate femtosecond Raman soliton pulses with the center wavelength continuously red-shifted by varying the input pulse energy. The pulse energy of a Raman soliton needs to satisfy the well-known soliton area theorem (Equation 4), which shows that the pulse energy is proportional to A_eff_, the fiber MFA. SFSS requires negative GVD to form Raman soliton, and therefore ultrafast Yb-fiber lasers cannot be used to excite SSFS in conventional single-mode fibers that exhibit positive GVD for wavelength <1.03 μm. Instead, PCFs with a small MFD (<3 μm) can offer negative GVD for the excitation pulses at ~1.03 μm. The associated strong nonlinearity in these PCFs limits the Raman soliton pulse energy to ~0.1 nJ. The conflict between negative GVD and large MFA in a single-mode fiber is resolved by higher-order-mode (HOM) fibers because they can exhibit negative GVD as well as have a large MFA. SSFS in HOM fibers allows generation of energetic Raman solitons with up to 80 nJ pulse energy pumped by Yb-fiber lasers (Rishøj et al., 2019, van Howe et al., 2007). Ultrafast Er-fiber lasers can be used to excite SSFS at the fundamental mode in LMA fibers. For example, using 1550-nm femtosecond pulses from an Er-fiber laser system as the excitation pulses, ~100-fs pulses tunable in 1,580–2,520 nm with up to 70 nJ pulse energy were obtained (Yu et al., 2019).

SESS constitutes a new fiber-optic wavelength conversion method featuring large wavelength tuning range (>400 nm) and superior energy scalability (Liu et al., 2016a, Liu et al., 2017a). SESS employs SPM-dominated nonlinearity in a short fiber to dramatically broaden an input narrowband optical spectrum to a broadband spectrum comprising well-isolated spectral lobes. A considerable portion of power is contained by the leftmost and the rightmost spectral lobes, which are selected by proper optical filters to produce nearly transform-limited femtosecond pulses (without external compression) with the center wavelength widely tunable. Excited in a PCF by an Yb-fiber ultrafast laser, SESS generated ~100-fs pulses tunable from 825 to 1,210 nm with >1 nJ pulse energy (Liu et al., 2016a). The SESS pulse energy can be scaled up by using shorter fiber with a larger MFA. Using 8-cm LMA fiber with 7.5-μm MFD, we demonstrated a SESS source that produced ~100-fs pulses tunable from 940 to 1,250 nm with up to 33 nJ pulse energy (Yu et al., 2019). To cover the two transmission windows (i.e., 1.3 and 1.7 μm) for biomedical microscopy imaging, we employed a high-power ultrafast Er-fiber MOPA system to excite SESS and achieved ~100-fs pulses tunable in 1.3–1.7 μm (Chung et al., 2017, Chung et al., 2018). We also showed that the SESS pulse energy can be scaled up to 130 nJ, leading to wavelength tunable pulses with >1 MW peak power (Chung et al., 2017). Using such a SESS source to drive multimodal multiphoton microscopy imaging, we have performed optical virtual biopsy in human skin (Chung et al., 2019a, Chung et al., 2019b).

## Conclusion and Outlook

Ultrafast fiber lasers undeniably constitute a powerful toolbox, which provides solution to various scientific and industrial problems. The versatility and capability of such toolbox relies heavily on the parameter space that ultrafast fiber lasers can access. In this paper, we reviewed recent progress in this fertile research field with a focus on improving laser parameters such as repetition rate, pulse energy, average power, pulse duration, and wavelength tuning range. A subset of these parameters defines the performance of an ultrafast fiber laser aiming for a specific application.

We restricted our discussion to ultrafast Yb-fiber and Er-fiber laser systems that operate at the near-infrared wavelength range. As Figure 1 indicates, ultrafast Tm-fiber and Ho-fiber lasers operating at ~2 μm have received increased research interest and progressed at a rapid pace. To generate femtosecond pulses at even longer wavelength directly from a laser, a passively mode-locked Er^3+^: fluoride glass fiber oscillator was demonstrated, which delivered 207-fs pulses at 2.8 μm (Duval et al., 2015). In addition to the emergence of new active fibers that bring forth ultrafast fiber lasers emitting at new wavelength (Zhu et al., 2017), remarkable advances appear in the development of passive fibers that are fabricated from new glass materials or designed with novel structures. We strongly believe that the continuous progress in both active and passive fibers will promote new techniques to further expand the toolbox of ultrafast fiber lasers, which will in turn solve the challenges faced by existing and emerging applications.

## Methods

All methods can be found in the accompanying Transparent Methods supplemental file.
